# How corporate entrepreneurship shapes millennials’ work engagement: the role of autonomy and competence

**DOI:** 10.3389/fpsyg.2026.1797168

**Published:** 2026-07-16

**Authors:** Smita Chaudhry, Amarpreet Singh Ghura

**Affiliations:** 1 Department of Human Resource Management, FLAME University, Pune, India; 2 Department of Entrepreneurship, FLAME University, Pune, India; 3 Department of Design, Indian Institute of Technology, Delhi, India

**Keywords:** autonomy, competency, corporate entrepreneurship, millennial generation, mixed methods, relatedness, self-determination, work engagement

## Abstract

**Introduction:**

Corporate entrepreneurship (CE) and work engagement (WE) are well-established topics in the literature; however, little is known about the psychological processes through which a CE ecosystem shapes employee engagement. Specifically, no prior studies have explained this relationship in terms of psychological need satisfaction. In addition, the millennial workforce, characterized by distinctive values and work preferences, has received limited attention in CE research. To address these gaps, this study applies self-determination theory (SDT) to examine how satisfaction of basic psychological needs accounts for the link between a CE ecosystem and millennials’ WE.

**Methods:**

We employed a convergent parallel mixed-methods design, independently and concurrently collecting primary data through semi-structured interviews with HR professionals from ten organizations in India and survey responses from 233 millennial employees within these organizations. The qualitative data were analyzed using thematic analysis, while the quantitative data were examined using regression, path, and effects analyses to test the proposed conceptual framework.

**Results:**

The findings indicate that a CE ecosystem is positively associated with millennials’ WE. This relationship is fully mediated by satisfaction of the needs for autonomy and competence, two core dimensions of SDT.

**Discussion:**

The results suggest that organizations seeking to improve engagement and retention among valued millennial employees may benefit from implementing CE ecosystems that are deliberately designed to satisfy these psychological needs, with positive implications for both individual and organizational performance. The study’s theoretical, empirical, methodological, and practical contributions are discussed.

## 1 Introduction

Work engagement has been extensively studied in scholarly literature due to its numerous benefits for both employees and organizations. It increases job satisfaction, organizational commitment, organizational citizenship behaviour, creativity, and task performance (Hui et al., 2021; Yin, 2018). It also lowers burnout, counterproductive work behaviour, and intentions to quit. Scholars have tried to understand the different organizational factors like workplace experience, meaningful work, workplace spirituality, collaborative environment, leadership, organizational support, job characteristics, and person-job fit that can affect engagement (Alnuaimi, 2022; Bantha et al., 2024; Haeckl and Rege, 2025; Hurtienne et al., 2022; Faiz Rasool et al., 2024; Pimenta et al., 2024; Sahni, 2021; Syalieta and Mas’ ud, 2025). In this paper, we seek to examine the relation of CE ecosystem with work engagement.

A CE ecosystem allows employees to seize internal entrepreneurial opportunities to innovate regardless of resources (Ireland and Kuratko, 2006; Kazanjian et al., 2017). It provides top management support, time for entrepreneurial activities, discretion in tasks and responsibilities, rewards for good performance, and clarity on expected outcomes (Kuratko et al., 2014), which can potentially motivate employees. Given the increased emphasis on CE to promote innovation and performance (Ghura and Erkut, 2024; Glinyanova et al., 2021), and the importance of engagement for organizational performance (Saks, 2019), it is imperative to understand how a CE ecosystem can promote work engagement.

Extant research examining the CE and engagement relationship remains limited in scope and theoretical breadth. Ahmed et al. (2020) demonstrated that CE promotes engagement as a pathway to business performance, whilst Kassa and Raju (2015) revealed that employees’ personal and professional appraisal of CE benefits drives their engagement. Whilst these studies provide foundational insights, they approach the CE–engagement relationship either through a business outcomes lens or an individual cost–benefit evaluation framework, neglecting the underlying psychological mechanisms that sustain employee motivation. We seek to address this gap.

In this paper, we examine the relationship between the CE ecosystem and work engagement through the lens of employees’ psychological need satisfaction. We use self-determination theory (SDT) for the purpose. SDT postulates that human beings have three fundamental psychological needs; autonomy, competence, and relatedness (Ryan and Deci, 2000). Satisfaction of these needs motivates them intrinsically and makes them feel fulfilled. In the course of their life, individuals strive to satisfy these needs to help them thrive (Chhajer and Chaudhry, 2022). SDT offers a nuanced understanding of sustained motivation grounded in fundamental psychological need satisfaction, making it a theoretically pertinent framework for examining enduring engagement (Gagné and Deci, 2005).

This study makes a significant theoretical contribution by being the first, to our knowledge, to examine the CE ecosystem - engagement relationship through the SDT lens. Whilst extant research has approached this relationship through business outcomes (Ahmed et al., 2020) or social exchange theory-based cost–benefit evaluation frameworks (Kassa and Raju, 2015), neither perspective adequately accounts for the intrinsic psychological mechanisms that sustain employee motivation and engagement within CE contexts. By applying SDT, this study establishes that the CE ecosystem engages employees not merely through extrinsic incentives or transactional reciprocity, but by systematically satisfying fundamental psychological needs for autonomy, competence, and relatedness, conditions that are structurally embedded within CE ecosystem dimensions. Specifically, work discretion and time availability address autonomy, performance-contingent rewards and top management support address competence, and flexible organisational boundaries and empathetic leadership address relatedness, collectively producing a need-satisfying motivational environment conducive to sustained intrinsic engagement (Ryan and Deci, 2000; Gagné and Deci, 2005). This theoretical integration advances the CE literature by reframing the CE ecosystem not merely as a strategic organisational tool for innovation and performance, but as a psychologically enriching work environment that engages employees through intrinsic motivation, a distinction with important implications for how organisations design and implement CE initiatives to sustain employee engagement.

We study the relationship between CE ecosystem and work engagement in the context of millennials. Existing studies have not considered the millennial workforce, despite established findings on the benefits of millennial entrepreneurship (Yiga et al., 2023). Millennials constitute a generation of people born between the early 1980s and mid-1990s. They are also referred to as Generation Y (Dimock, 2018), and comprise the majority of the global working population. As per generational cohort theory (Bettelheim, 1962), every generation has its own set of shared values, attitude and behaviours, which can affect how a CE ecosystem may impact engagement. Millennials seek purpose, worthwhile employment, and participation in meaningful work (Forastero et al., 2018). They like to tackle a project in ways that may be considered unconventional. They prefer tasks offering self-directed learning, quick feedback, flexibility, freedom, and career advancement (Wiedmer, 2015). These values and preferences of millennials are aligned with corporate venturing activities (Guerrero et al., 2021), and can affect engagement (Schullery, 2013).

By focusing on millennial employees, we also extend the generational cohort theory (Trzesniewski and Donnellan, 2010). Given millennials’ preference for meaningful, dynamic, and growth-oriented work (Hartman and McCambridge, 2011), they may be particularly responsive to CE ecosystem initiatives, suggesting that CE functions as a generation-specific engagement mechanism. Furthermore, CE ecosystem offers unique potential for engaging millennials in certain professions characterised by limited innovation and restricted career progression, adding contextual nuance to existing generational engagement literature (Ghura et al., 2022).

We conducted the study using a convergent parallel mixed-method (CPMM) design involving simultaneous collection, analysis and interpretation of data through two different methods and two different respondent profiles. We collected qualitative data through semi-structured interviews with HR heads of organizations, and quantitative data through field surveys with employees. The design helped to understand the convergence and complementarity of the results obtained from the organizational and employees’ perspectives. The respondent sample for both the qualitative and quantitative data was from India.

The study contributes to the literature in three ways. First, it advances our understanding of the importance of SDT (in terms of satisfaction of fundamental psychological needs) within the CE ecosystem. We do not know of any studies that have investigated the association between CE ecosystem and work engagement from the standpoint of psychological needs satisfaction. Second, it explores work engagement in a CE ecosystem, in the specific context of millennials, considering their unique characteristics, values and preferences. SDT is especially relevant in the context of millennials, as the need for autonomy, competence, and relatedness is more pronounced in them due to their distinctive characteristics (Ng and Johnson, 2015). Third, it investigates the conceptual model using mixed methods, which helps cross-verify and validate the results obtained from two different types of data (qualitative and quantitative), two different samples (HR managers and employees), and two different perspectives (organization and employees).

## 2 Theory and hypotheses

### 2.1 Work engagement and millennials

Kahn (1990) defines work engagement as employees’ willingness to physically, cognitively, and emotionally embrace their work. Work engagement includes employees’ interest, satisfaction, and enthusiasm towards work, involvement in organization’s operations, and adoption of its values (Robinson, 2006). While conceptualization by Kahn (1990)emphasizes the psychological conditions that enable personal work engagement, subsequent scholars have extended this view to capture behavioural and affective dimensions (Schaufeli et al., 2003). However, existing definitions have been developed predominantly in traditional organizational contexts, leaving open the question of how engagement operates within entrepreneurial or innovation-oriented environments, a gap this study addresses by situating the CE ecosystem as the antecedent context.

Extant studies have discussed the characteristics of millennials that set them apart from other generations. Millennials are self-assured and independent, have high self-esteem, and expect a good quality job (Trzesniewski and Donnellan, 2010). They give importance to company values. They are attracted by transparency, enthusiasm, organizational trust and collaboration. Since millennials are more confident of their problem-solving skills, they dislike fixed working hours and prefer freelancing even if it pays less (Ghura and Goel, 2018). Such characteristics can prohibit millennials from being engaged, fulfilled, and committed to their employment. Most jobs require employees to use their qualifications and skills to accomplish tasks in a pre-defined pattern and rarely explain their effect on organizational outcomes. As a result, millennials may find the processes too formal, the environment not dynamic enough, and work meaningless, thus leading to poor engagement and high turnover (Hartman and McCambridge, 2011; Purba and Ananta, 2018).

Despite substantial documentation of millennial disengagement risks, prior studies have not theorized why specific organizational environments (e.g., a CE ecosystem) may mitigate these risks. The literature also conflates demographic descriptions with engagement antecedents, stopping short of identifying the psychological mechanisms through which millennial characteristics translate into engagement or disengagement. The present study fills this gap by highlighting these mechanisms, thereby indicating the relevance of millennials to the study’s conceptual model.

### 2.2 CE ecosystem

CE ecosystem refers to the organizational environment that facilitates corporate entrepreneurship. “Corporate entrepreneurship is a process by which individuals in a current organization undertake entrepreneurial opportunities to innovate regardless of the level and nature of resources” (Ireland and Kuratko, 2006, pp. 10). The CE ecosystem comprises the resources, information and support from the organization to enable employees to embrace CE, and it indicates the organizational preparedness for CE. Corporate entrepreneurs innovate by collaborating, and utilizing opportunities to improve products and processes. The Corporate Entrepreneurship Assessment Instrument (CEAI) indicates the organizational factors that constitute the CE ecosystem (Kuratko et al., 2014).

CEAI comprises five dimensions. Top management support pertains to all kinds of support from the top management to bring about entrepreneurial behaviour. Work discretion refers to the liberty to make decisions about individual work with minimum oversight. Rewards pertain to the awards for entrepreneurial achievement. Time availability pertains to time for experimentation and innovation to fulfill organizational goals. Organizational boundaries pertain to a clear understanding of organizational work outcomes, and transparency in assessing, identifying and executing entrepreneurial activities.

Management scholars have found favorable outcomes for organizations that practice CE. CE leads to renewed organizational strategies, and improved growth and profitability and innovations (Glinyanova et al., 2021; Tseng and Tseng, 2019). It makes organisations more proactive and enhances their risk-taking ability (Kuratko et al., 2014), enabling organizational performance (Bierwerth et al., 2015). From the employee standpoint, CE positively affects knowledge and skill development (Ireland and Kuratko, 2006).

#### 2.2.1 CE ecosystem and millennials’ work engagement

The Job Demands-Resources (JD-R) theory (Demerouti et al., 2001) provides a theoretical lens for understanding the relation of CE ecosystem with work engagement. JD-R theory posits that job resources foster engagement through a motivational process whereby adequate resources satisfy fundamental psychological needs, facilitate goal achievement, and stimulate personal growth. The five CE ecosystem dimensions collectively constitute the organisational job resources that activate this motivational process, thereby fostering sustained millennial work engagement. Top management support provides employees with strategic guidance, mentoring, and institutional backing, reducing the psychological uncertainty inherent in entrepreneurial work whilst activating intrinsic motivation (Kuratko et al., 2014). Work discretion constitutes an autonomy-related resource that empowers employees to self-direct entrepreneurial activities, translating organisational entrepreneurial intent into personally meaningful and engaging work experiences (Schaufeli and Bakker, 2004). Rewards serve as performance-contingent informational resources that affirm employee effectiveness and reinforce the motivational process by strengthening employees’ belief in the instrumentality of CE and engagement (Deci et al., 1999). Time availability buffers the health-impairment process by mitigating the debilitating effects of workload pressure, creating cognitive and temporal space for deep entrepreneurial engagement without risking burnout (Amabile et al., 1996). Flexible organisational boundaries reduce structural barriers impeding entrepreneurial initiative, enabling employees to mobilise cross-functional knowledge and collaborative resources necessary for sustained participation (Ireland et al., 2009). Thus, all five dimensions are likely to promote work engagement.

Some studies have indicated a positive relation of CE with engagement (Ahmed et al., 2020; Kassa and Raju, 2015). Ahmed et al. (2020) established a positive association between CE and engagement but treated engagement as an outcome of organizational-level exchanges, without examining the intra-individual psychological pathways. Similarly, Kassa and Raju (2015) linked CE to engagement via perceived support. However, neither study explained which specific CE dimensions activate engagement or through what psychological mechanism engagement occurred. Crucially, neither study examined this relationship for a generationally specific workforce. This leaves a critical theoretical gap: existing evidence is correlational in nature and does not explain the psychological processes linking CE ecosystem dimensions to engagement, nor does it account for how the distinct values of millennials shape this pathway. The present study addresses this gap by introducing SDT as the explanatory mechanism. Specifically, it proposes that the five CE ecosystem dimensions (top management support, work discretion, rewards, time availability, and organizational boundaries) satisfy the millennials’ psychological needs, thereby producing engagement.

We propose that the impact of the CE ecosystem on engagement is likely to be more pronounced in the context of the millennial workforce, due to their characteristic values and preferences (Ghura, 2017; Ghura and Goel, 2018). Millennials are inclined towards work that evokes their passion and creativity, has purpose, offers multitasking and flexibility, allows problem-solving and transparency, is reasonably risky, develops competency, and promotes teamwork and trust (Wiedmer, 2015). They value learning, problem-solving, experimenting, and taking calculated risks. They are inclined towards leisure rewards, higher perks, advancement opportunities and work-life balance, compared to earlier generations (Brant and Castro, 2019). Many of these work preferences of millennials align with the attributes of CE (Ireland and Kuratko, 2006).

Millennials represent a theoretically appropriate population for examining the CE ecosystem - engagement relationship, as their characteristic values and preferences, including desire for autonomy, mastery, experimentation, and meaningful collaborative work, align closely with the motivational conditions that CE ecosystems are designed to cultivate (Wiedmer, 2015; Brant and Castro, 2019). Therefore, we propose:

*H1*: An organization’s CE ecosystem has a positive effect on the millennial employees’ work engagement.

### 2.3 Role of self-determination theory (SDT)

SDT is a motivational theory that postulates that individuals perform well if they receive psychological fulfilment at work (Ryan and Deci, 2000). According to SDT, humans have the fundamental psychological needs for autonomy, competence, and relatedness (Ryan and Deci, 2000). Satisfaction of these needs boosts their performance, by increasing job satisfaction (Kovjanic et al., 2012), task motivation, psychological adjustment (Ryan and Deci, 2000), thriving (Chhajer and Chaudhry, 2022; Spreitzer and Porath, 2014) and well-being (Slemp and Vella-Brodrick, 2013). SDT helps organizations identify how they can intrinsically motivate employees in ways that benefit both employees and them, for example, through job crafting (Slemp and Vella-Brodrick, 2013). Previous studies have indicated the relevance of SDT in explaining employee engagement (Meyer and Gagne, 2008) and intentions for entrepreneurial activity (Chung and Lee, 2020). However, SDT-related studies in the context of CE are limited.

Whilst the CE ecosystem has been predominantly examined through an organisational performance lens, its psychological implications for employee motivation remain comparatively underexplored. SDT contends that the social and organisational environment plays a critical role in either nurturing or frustrating the fundamental psychological needs, thereby determining the quality of individual motivation and work engagement (Ryan and Deci, 2000). Thus, the CE ecosystem constitutes precisely such an organisational environment that at the theoretical level can systematically address all three SDT psychological needs simultaneously, making it a particularly relevant contextual framework for understanding millennial work engagement.

The importance of SDT for millennials is established in previous studies (Nwoko and Yazdani, 2023). The relevance of millennials to the CE ecosystem – SDT- engagement research model is structurally embedded within each hypothesised pathway. Millennials are disproportionately responsive to CE ecosystem dimensions such as work discretion, time availability, and top management support, which directly address their psychological needs for self-direction, competence development, and organisational belonging, making SDT a particularly apposite theoretical lens for understanding how CE ecosystems engage this specific generational cohort (Ireland and Kuratko, 2006; Ryan and Deci, 2000).

#### 2.3.1 Role of autonomy

Autonomy denotes the innate power to exercise will and have choice and freedom in actions and interactions with the environment. It can also be defined as volition or a feeling of being in control of one’s actions (Ryan and Deci, 2000). A CE ecosystem is intended to provide a supportive and flexible environment to promote employee entrepreneurial behaviour. According to organizational support theory (Eisenberger et al., 1986), factors like top management support, work discretion, rewards, time availability and organizational boundaries signal encouragement and trust in the employees, which make them feel empowered in making decisions and executing tasks. In particular, top management support contributes to autonomy satisfaction by signalling organisational endorsement of employee-initiated entrepreneurial activities, reducing the psychological risk associated with autonomous decision-making (Hornsby et al., 2002). Work discretion affords employees the freedom to make independent decisions, exercise personal judgment, and pursue entrepreneurial initiatives without excessive supervisory oversight (Kuratko et al., 2014). This latitude for self-directed action is fundamentally autonomy-supportive, enabling employees to align work behaviour with intrinsic values rather than external demands. Flexible organisational boundaries further reinforce autonomy by reducing bureaucratic constraints and tolerating experimentation, creating conditions where employees can pursue innovative ideas across functional boundaries (Ireland et al., 2009). Collectively, these dimensions create an autonomy-supportive environment that encourages volitional, self-determined engagement with CE initiatives.

Literature shows that autonomy (as an aspect of SDT) is associated with engagement (Meyer and Gagne, 2008). Autonomy empowers employees to behave freely within their professional roles and boosts engagement (Lee, 2012). They have fewer obstacles, less conflict, and more control. This improves performance and dedication. Job choices further assist the employees reach their goals.

Millennials’ strong preference for self-direction, risk-taking, and freedom from hierarchical oversight (Sarah, 2007) renders them disproportionately responsive to the autonomy-supportive conditions generated by CE ecosystem dimensions. Specifically, work discretion and time availability directly address millennials’ expectations of comprehensive self-determination by affording decision-making latitude, freedom to experiment, and tolerance for failure. These conditions are generationally salient for a millennials cohort that prioritises volitional self-expression and entrepreneurial ownership over compliance with managerial directives (Hartman and McCambridge, 2011). The multidimensional autonomy afforded by CE ecosystems, encompassing functional, decision, structural, and strategic autonomy (Gard et al., 2013), maps comprehensively onto millennials’ holistic expectation of self-determination across all aspects of their work role.

Thus, we propose that the CE ecosystem would empower the millennial employees to experiment with their ideas and make meaningful impact with easy access to resources and time. Given the values and preferences of millennials, this would satisfy their need for autonomy, and thereby promote engagement.

*H2*: The relation between an organization’s CE ecosystem and millennial employees' work engagement is mediated by the satisfaction of their need for autonomy.

#### 2.3.2 Role of competence

Competence implies being capable of handling the environment. Competence is demonstrated when individuals internalize essential knowledge, skills and abilities to perform activities in the most effective manner. A CE ecosystem is intended to encourage learning, experimentation, and development of employees, for the organization to adapt and thrive in a rapidly changing environment. As per dynamic capabilities theory (Teece et al., 1997), these opportunities develop competence in the employees to become more innovative and agile. In particular, top management support, manifested through mentoring, resource provision, and strategic guidance, equips employees with the knowledge and organisational backing necessary to successfully execute entrepreneurial initiatives, reinforcing perceptions of effectiveness and capability (Kuratko et al., 2014). Time availability enables deliberate exploration, skill development, and iterative problem-solving without the cognitive pressure of competing demands, creating optimal conditions for competence growth (Amabile et al., 1996). Performance-contingent rewards that recognise entrepreneurial achievements, serve as critical informational feedback affirming employees’ capability. When perceived as acknowledging competence rather than controlling behaviour, such rewards sustain intrinsic motivation and deepen engagement (Deci et al., 1999). Furthermore, well-designed organisational boundaries that define the scope of entrepreneurial activity provide employees with clear performance parameters within which competence can be meaningfully demonstrated and evaluated (Ireland et al., 2009). Together, these dimensions create a competence-nurturing environment that align with SDT’s assertion that feelings of effectiveness and mastery are fundamental drivers of sustained intrinsic motivation (Ryan and Deci, 2000).

Literature shows that competency (as an aspect of SDT) is associated with engagement (Meyer and Gagne, 2008). Competence keeps employees interested in work by enabling them to try out and use new approaches to get work done. It makes them feel more capable and efficacious about overcoming challenging tasks and thus achieve their professional goals (Fetriah and Hermingsih, 2023).

Millennials’ strong orientation towards mastery, learning, and embracing challenging tasks (Brant and Castro, 2019) makes them particularly receptive to the competence-building mechanisms of CE ecosystems. Assignment-based entrepreneurial challenges, mentoring through top management support, and performance-contingent rewards directly address millennials’ desire to develop capabilities, demonstrate effectiveness, and receive meaningful recognition. These conditions satisfy competence needs in ways uniquely aligned with this generation’s professional aspirations.

Thus, we propose that the CE ecosystem would provide the resources, opportunities, time and incentives to build desirable skills and capabilities in the millennial employees. Given the values and preferences of millennials, this would satisfy their need for competence, and thereby promote engagement.

*H3*: The relation between an organization’s CE ecosystem and millennial employees' work engagement is mediated by the satisfaction of their need for competence.

#### 2.3.3 Role of relatedness

Relatedness implies feeling socially connected to others. It results in trusting, respectful, and caring relationships. A CE ecosystem is expected to provide resources, incentives and opportunities to employees to meet the shared organizational goal of competitiveness in the marketplace. As per social interdependence theory (Deutsch, 1949), employees make efforts to connect and collaborate with others due to the structural interdependence of their roles and expected synergy from the collaboration. In particular, top management support, characterised by genuine interpersonal investment like empathetic leadership, active listening, and visible advocacy for employee entrepreneurial efforts, communicates that employees are valued organisational members, directly satisfying the need to feel cared for and connected (Ryan and Deci, 2000). This sense of being supported by organisational leadership fosters psychological safety, a prerequisite for the risk-taking and collaborative behaviour inherent in CE activities. Permeable organisational boundaries that encourage cross-functional collaboration and collective entrepreneurial endeavour strengthen interpersonal connectedness, fostering a shared sense of entrepreneurial community and belonging (Hornsby et al., 2002). Team-oriented rewards structures that recognise collective entrepreneurial contributions reinforce cooperative norms and collective belonging, satisfying relatedness needs whilst simultaneously incentivising CE participation (Kuratko et al., 1990). Time availability further supports relatedness by creating space for collaborative ideation and relationship-building activities that are frequently sacrificed under excessive workload conditions. Collectively, these dimensions produce a relatedness-supportive environment consistent with SDT’s proposition that interpersonal connectedness and belonging are fundamental to sustained motivational engagement (Ryan and Deci, 2000).

Literature shows that relatedness (as an aspect of SDT) is associated with engagement (Meyer and Gagne, 2008). Relatedness makes employees exchange thoughts and perspectives. It also provides identification and sense of belonging. Conversations build trust and enable good quality decisions. The shared experiences and social connections increase interest and involvement in work.

Millennials’ desire for belongingness, trust-inducing relationships, and collaborative work (Wiedmer, 2015) positions them as inherently responsive to the relatedness-supportive conditions of CE ecosystems. Permeable organisational boundaries, team-oriented rewards, and empathetic top management support, collectively create the interpersonal connectedness and organisational belonging that millennials actively seek, theoretically establishing relatedness as a meaningful mediating condition between CE ecosystem and millennial engagement.

Thus, we propose that the CE ecosystem would promote the requirement and opportunity for socially embedded learning and build social capital amongst millennial employees. Given the values and preferences of millennials, this would satisfy their need for relatedness, and thereby promote engagement.

*H4*: The relation between an organization’s CE ecosystem and millennial employees' work engagement is mediated by the satisfaction of their need for relatedness.

## 3 Materials and methods

We employed a convergent parallel mixed-method (CPMM) design to investigate the effect of the CE ecosystem on millennials’ engagement (Onwuegbuzie and Collins, 2007). CPMM allows simultaneous analysis and interpretation of data collected through qualitative and quantitative methods (Creswell and Clark, 2007). We used the qualitative method of semi-structured interviews to collect data from HR heads of organizations that have established a CE ecosystem in their organization. We used the quantitative method of survey questionnaires to collect data from the millennial employees. Since data had to be collected from human participants, we got approval from our university’s IRB committee (IRB approval number 2022/08/01 EXP) before proceeding with the research.

The adoption of a CPMM design was epistemologically and methodologically warranted for several reasons. First, the inherent complexity of the research phenomenon necessitated a mixed-method approach. Since the CE ecosystem represents an organisational-level initiative whilst WE reflects an individual employee attitude, examining the CE–WE relationship through a single methodological lens would have produced an incomplete understanding of a phenomenon that operates simultaneously at organisational and individual levels. The mixed-methods design addressed this by capturing HR managers’ organisational perspective through semi-structured interviews and millennial employees’ experiential perspective through survey questionnaires, enabling a holistic examination of the research problem (Molina-Azorín et al., 2012). Second, collecting qualitative and quantitative data from distinct respondent groups using different instruments provided a methodological safeguard against common method bias, substantially reducing the risk of systematic response bias attributable to a single data source (Podsakoff et al., 2003). Third, the design enabled verification of the theoretical significance of SDT constructs (autonomy, competence, and relatedness) from both organisational and employee perspectives. The alignment between what organisations perceive as important CE-driven engagement mechanisms and what employees actually experience bridges the often-cited gap between managerial intentions and employee outcomes, strengthening the practical applicability of findings and offering organisations evidence-based guidance for leveraging CE ecosystems to foster millennial engagement (Creswell and Clark, 2007).

The CPMM design capitalised on the complementary strengths of qualitative and quantitative methods whilst mitigating their respective limitations (Molina-Azorín et al., 2012). The qualitative strand provided contextual depth and theoretical richness by capturing HR managers’ experiential accounts of CE ecosystem implementation, whilst the quantitative strand enabled statistical rigour, hypothesis testing, and generalisability across a larger sample of millennial employees. The integration of both strands produced a more robust understanding of the CE and WE relationship than either method could have achieved independently. Specifically, triangulation cross-validated quantitative findings derived from millennial employees’ self-reported perceptions of CE ecosystem, SDT needs, and WE against qualitative insights from HR managers’ organisational perspectives (Onwuegbuzie and Collins, 2007). Where both strands converged, the hypothesised CE–SDT–WE relationships gained stronger empirical support; where divergence emerged, qualitative data provided contextual explanations that enriched the interpretation of quantitative results. This reciprocal relationship is particularly valuable where subjective employee perceptions and objective managerial observations of the same phenomenon may diverge considerably, mitigating the risk of context-specific or methodologically artefactual findings (Molina-Azorín et al., 2012).

### 3.1 Qualitative method

Using purposive sampling, we reached out to 18 organizations in India, which have a CE ecosystem for their employees, and received informed consent from 10 organizations. Purposive sampling was used to identify organizations that had explicitly introduced CE initiatives and employed a substantial number of millennial employees. This approach ensured information-rich cases but may over-represent organizations that are relatively advanced in their CE practices and more willing to discuss them. To partially mitigate this potential bias, we targeted organizations from multiple industries and of varying sizes, and we stopped recruitment when additional interviews yielded only marginal new information, indicating thematic saturation.

These organizations appointed their human resource head as the respondent for our semi-structured interviews. Each respondent had directly or indirectly been involved in managing the CE ecosystem, and had experience in dealing with millennial employees. Among the participants interviewed (*n* = 10), 40% were female. Maintaining the internal validity of the study, a strict protocol was followed where in same set of questions was asked to each participant (Ghura and Erkut, 2024). We conducted the interviews from February 2023 to April 2023. All interviews were conducted in English and lasted for almost 50 min and were audio recorded, and consequently transcribed with the permission of the participants. When participants were adding only marginal contribution to increase the knowledge they were not asked anything further.

The target population for the qualitative phase comprised HR heads or senior HR managers in medium-to-large organizations in India that had an explicitly articulated CE ecosystem and employed a sizeable millennial workforce. The final qualitative sample consisted of 10 HR heads (4 women and 6 men) from organizations operating in IT services, manufacturing, financial services, fast-moving consumer goods and consulting, with organizational sizes ranging from approximately 500 to over 5,000 employees (Table 1). These organizations were located in major Indian metropolitan regions (e.g., Delhi-NCR, Mumbai, Bengaluru), which are hubs for millennial employment in CE-oriented firms.

**Table 1:** Profile of interview participants.

S. no	Company sector	Interview length	Percentage of millennials in the workforce
1	Biopharmaceuticals	29 min	40%
2	Energy conservation	28 min	10%
3	Marketing technology service	26 min	40%
4	Capital goods packaging	31 min	70%
5	FMCG	27 min	30%
6	Cement manufacturing	24 min	50%
7	Infrastructure	25 min	60%
8	IT consulting and software outsourcing	34 min	55%
9	Health (pharma supplements)	25 min	30%
10	IT services and consulting	29 min	60%

Interviews were imported to NVivo12 software. Researchers read and re-read the transcripts to create broad-based categories. The interviews were thematically analyzed using a provisional code list (first-order categories) developed from CE dimensions (Ridder, 2016). After identifying the concepts in the data, the data was segmented in small units (the coding units). Sentences were used as the coding unit. It acted as the segmentation criterion and was used throughout the coding phase. The coding scheme used is theory-driven. Text about the CE ecosystem’s impact was identified and mapped to the CE dimensions. This text was further classified under second-order themes of autonomy, competence and relatedness. In accordance with recommendation by Boyatzis (1998), a codebook was created that included names for labels, themes of focus, when themes occur, criteria for inclusion and exclusion, and examples.

In NVivo, each first-order category and second-order theme was created as a node, and all relevant segments were coded to these nodes. Coding was iterative: two researchers independently coded the full data set, compared codes, and resolved discrepancies through discussion, which led to refinement of node definitions and inclusion–exclusion criteria. For each node, NVivo coding queries were used to extract the number of respondents contributing to that theme and the frequency of coded references, which guided the identification of dominant themes reported in the results section.

To confirm the reliability of our interpretations, we conducted two member checks. This involved taking the emerging pattern-seeking sketch back to the site and having the interview participants examine it closely. The purpose was to confirm that the interpretive framework was “making sense to, and validated by those experiencing the phenomena of interest” (Nag et al., 2007). The interviews produced explicit self-theories, and insights derived from the reporting of incidents, arguments, and descriptions. For data-triangulation purposes, the information provided by the participants during the interview was matched with the information available on the organisation website. Data sources such as press and media reports was used in order to increase the validity of our data and to corroborate the facts stated by the participants on organisation age, size, and their initiatives related to CE ecosystem and its impact on millennials’ engagement.

A semi-structured interview guide was used to ensure comparability across interviews while allowing participants to elaborate on issues most relevant to their organisational context (see Appendix). The guide included open-ended questions about (a) the nature and components of the CE ecosystem in the organization, (b) perceived effects of CE practices on millennial employees’ motivation, performance and engagement, and (c) specific ways in which CE initiatives influenced millennials’ sense of autonomy, competence and relatedness. Probing questions were used to obtain concrete examples and to clarify how CE policies and practices were implemented in day-to-day work.

### 3.2 Quantitative method

We prepared a questionnaire survey to collect quantitative data. The survey consisted of established scales identified from the literature for all constructs. The first step was to validate the survey to ensure that it accurately and reliably measured the constructs. We performed content validation to assess the items on relevance, clarity, and comprehensiveness, with the help of two academic experts. We then refined the survey further by taking feedback from two millennial employees (not part of the final sample) on their understanding of the items and their relationship with the constructs, and to revise ambiguous or misunderstood items, thus improving the face validity of the survey. The survey was found to be valid. No items were deleted.

The target population for the quantitative phase comprised millennial employees (born between early 1980s and mid-1990s) working in organizations in India that had established CE initiatives. The final sample of 233 millennials included at least 80 female and at least 150 male employees, with 3 respondents choosing not to disclose gender. The sample represented a range of education levels (e.g., undergraduate, postgraduate, professional degrees) and functional areas (such as operations, marketing, R&D, HR and finance). In terms of organizational hierarchy, 21% of respondents were at the entry level, 69.1% at the middle level and 9.9% at the senior level, which broadly reflects the pyramid structure of the participating organizations’ millennial workforce.

We collected quantitative data through snowball and purposeful sampling. HR heads first identified millennial employees who were directly exposed to CE initiatives, and these employees then referred colleagues who met the inclusion criteria. While this approach facilitated access to a hard-to-reach population across several organisations, it may introduce sampling bias because respondents are connected through professional networks and may share similar attitudes towards CE. To reduce this risk, we requested HR heads to nominate employees from different departments and hierarchical levels and capped the number of referrals from any single employee, thereby broadening the diversity of perspectives in the sample.

We also reached out to some millennials working in the other eight organizations whom we could not include for qualitative data collection. We informed the employees about the objectives of the study, and sent the google form survey. We conveyed to them that participating in the study was optional, and assured them about the confidentiality of their responses. No names were collected through the survey, thus mitigating social desirability and acquiescence bias. The respondents had to provide responses about their perception of the CE ecosystem, satisfaction of the need for autonomy, competence and relatedness and their engagement, on a 7-point Likert scale (ranging from 1—*Strongly Disagree* to 7—*Strongly Agree*).

We collected 251 complete responses. Verification of the age group resulted in removal of 18 responses that belonged to X and Z generation employees. The consequent sample size was 233. It comprised at least 80 female employees and at least 150 male employees (3 employees preferred not to reveal their gender). 21% of employees were at the entry level, 69.1% were at the middle level and 9.9% were at the senior level.

We conducted analysis using IBM SPSS version 28, SPSS PROCESS macro and IBM AMOS version 28 (for structural equation modelling). Exploratory factor analysis (EFA) helped to remove items that had a factor loading less than 0.5, or significant cross-loadings across factors. Confirmatory factor analysis (CFA) helped to further remove items that had unacceptable regression weights. The remaining items were selected based on Cronbach’s Alpha (*α*) reliability ≥0.70. Composite reliability (CR), convergent validity (CV) and discriminant validity (DV) of the variables were also estimated using average variance extracted (AVE) and inter-construct correlations (Fornell and Larcker, 1981). AVE indicates the percentage of variance in a variable caused by the corresponding items, and is calculated using their factor loadings. H1 was tested using hierarchical regression and all mediation hypotheses were tested using path analysis, and verified with effects analysis (Preacher and Hayes, 2004).

We conducted a robustness check of the study results by testing our assumptions about outliers, normality, heteroskedasticity *and m*ulticollinearity. We checked for multivariate outliers by observing Mahalanobis distance (Mahalanobis *d*^2^), and did not find any evidence of outliers. Normality of all model variables was examined using skewness and kurtosis statistics (*N* = 233). CE ecosystem (Sk = −0.85, Ku = 0.73), need for autonomy (Sk = −1.27, Ku = 1.62), need for competence (Sk = −1.19, Ku = 2.19), need for relatedness (Sk = −1.32, Ku = 1.89), and work engagement (Sk = −0.81, Ku = −0.15) recorded skewness values within the acceptable ±2 range and kurtosis values well within the ±7 threshold (George and Mallery, 2024), confirming the normality assumption. To test heteroskedasticity, we conducted White test. Insignificant results [*χ^2^*(213) = 225.47, ns] indicated that the error variance did not change with the values of CE. We checked for multicollinearity by finding out the variance inflation factor (VIF). The maximum VIF was 3.2, indicating that CE and SDT dimensions were not co-linear.

Common method bias was assessed using Harman’s single-factor test (Harman, 1976). All model variables were subjected to an unrotated principal component analysis (PCA). The results revealed six factors with eigenvalues exceeding 1.0, collectively explaining 66.19% of the total variance. The first single factor accounted for 43.68% of the total variance, falling below the 50% threshold, thus suggesting that common method bias is unlikely to pose a significant threat to the validity of the study’s findings.

#### 3.2.1 Measures

To measure the CE ecosystem, we adapted an 18-item scale by Hornsby et al. (2013). Sample item is “I have the freedom to decide what I do on my job.” EFA and CFA led to selection of 16 items for CE (*α* = 0.93, CR = 0.94, AVE = 0.51).

Measures for satisfaction of the need for autonomy (NAT) (seven items), competence (NCM) (six items) and relatedness (NRL) (10 items) were adapted from the scales used by Van den Broeck et al. (2010). Sample items for the respective measures are “I feel like I can be myself at my job”, “I am good at the things I do in my job”, and “At work, I can talk with people about things that really matter to me”. EFA and CFA led to selection of four items for autonomy (*α* = 0.88, CR = 0.92, AVE = 0.74), four items for competence (*α* = 0.81, CR = 0.88, AVE = 0.65) and five items for relatedness (*α* = 0.84, CR = 0.89, AVE = 0.62). Principal component analysis using varimax rotation revealed three factors, with each factor corresponding to the selected items for each variable.

We adapted the measure for work engagement (WE) from the 9-item scale used by Schaufeli et al. (2003). EFA and CFA led to selection of 7 items for WE (α = 0.91, CR = 0.94, AVE = 0.67). For all the measures, AVE was more than 0.5 indicating CV (Fornell and Larcker, 1981).

The factor loadings for the selected items for all measures are provided in Table 2a. The goodness-of-fit results of CFA are provided in Table 2b. The covariance analysis for the CFA is provided in Table 2c.

**Table 2a:** Item factor loadings for the constructs of the study.

Corporate entrepreneurship ecosystem		Factor loading
CE1	I have the freedom to decide what I do on my job.	0.80
CE2	It is basically my own responsibility to decide how my job gets done.	0.69
CE3	I have much autonomy on my job and am left on my own to do my own work.	0.73
CE4	I feel that I am my own boss and do not have to double-check all of my decisions with someone else.	0.69
CE5	I have just the right amount of time and workload to do everything well.	0.60
CE6	I always have plenty of time to get everything done.	0.68
CE7	My co-workers and I always find time for long term problem solving.	0.63
CE8	People are often encouraged to take calculated risks with ideas around here.	0.74
CE9	This business unit supports many small and experimental projects realizing that some will undoubtedly fail	0.75
CE10	Senior managers encourage innovators to bend rules and rigid procedures in order to keep promising ideas on track.	0.79
CE11	Those employees who come up with innovative ideas on their own often receive management encouragement for their activities	0.76
CE12	Money is often available to get new ideas off the ground.	0.80
CE13	My supervisor will give me special recognition if my work performance is especially good.	0.75
CE14	My manager will tell his/her boss if my work was outstanding.	0.74
CE15	The rewards I receive are dependent upon my work on the job.	0.67
CE16	My job description clearly specifies the standards of performance on which my job is evaluated	0.53
CE17	I seldom have to follow the same work methods or steps for doing my major tasks from day to day (removed item)	<0.50
CE18	In the past few months, I have always followed standard operating procedures or practices (removed item)	<0.50
Self-determination theory dimensions		
NAT1	I feel free to express my ideas and opinions in this job	0.88
NAT2	I feel like I can be myself at my job	0.87
NAT3	The tasks I have to do at work are in line with what I really want to do	0.83
NAT4	I feel free to do my job the way I think it could best be done	0.85
NAT5	At work, I often feel like I have to follow other people’s commands (removed item)	<0.50
NAT6	If I could choose, I would do things at work differently (removed item)	<0.50
NAT7	In my job, I feel forced to do things I do not want to do (removed item)	<0.50
NCM1	I really master my tasks at my job	0.85
NCM2	I feel competent at my job	0.83
NCM3	I am good at the things I do in my job	0.84
NCM4	I have the feeling that I can even accomplish the most difficult tasks at work	0.68
NCM5	I do not really feel competent in my job (removed item)	<0.50
NCM6	I doubt whether I am able to execute my job properly (removed item)	<0.50
NRL1	At work, I feel part of a group	0.77
NRL2	At work, I can talk with people about things that really matter to me	0.85
NRL3	At work, people involve me in social activities	0.76
NRL4	At work, there are people who really understand me	0.81
NRL5	Some people I work with are close friends of mine	0.74
NRL6	I do not really feel connected with other people at my job (removed item)	<0.50
NRL7	I do not really mix with other people at my job (removed item)	<0.50
NRL8	I often feel alone when I am with my colleagues (removed item)	<0.50
NRL9	At work, no one cares about me (removed item)	<0.50
NRL10	There is nobody I can share my thoughts with if I would want to do so (removed item)	<0.50
Work engagement		
WE1	I am enthusiastic about my job.	0.84
WE2	I feel happy when I am working intensely.	0.76
WE3	At my job, I feel strong and vigorous.	0.86
WE4	My job inspires me.	0.88
WE5	I am immersed in my work.	0.82
WE6	When I get up in the morning, I feel like going to work.	0.80
WE7	I am proud of the work that I do.	0.77
WE8	At my work, I feel bursting with energy (removed item).	<0.50
WE9	I get carried away when I am working (removed item).	<0.50

*N* = 233.

**Table 2b:** Goodness of fit results for CFA.

CFA model	Overall fitment	CMIN/df	RMSEA	SRMR	CFI	IFI
Goodness of fit results	*χ*^2^ (541) = 1020.23 (*p* > 0.05)	1.89	0.06	0.05	0.92	0.92

*N* = 233. RMSEA, root mean square error of approximation; SRMR, standardized root mean square residual; CFI, comparative fit index; IFI, incremental fit index.

**Table 2c:** Covariance results for CFA.

Covariances			Estimate	Standard error	Critical ratio	Significance
CE	<-->	WE	0.768	0.109	7.029	***
CE	<-->	NAT	0.839	0.109	7.726	***
CE	<-->	NCM	0.469	0.078	5.978	***
CE	<-->	NRL	0.709	0.116	6.089	***
NAT	<-->	NRL	0.593	0.093	6.354	***
NAT	<-->	NCM	0.415	0.064	6.521	***
NCM	<-->	NRL	0.350	0.067	5.193	***
NAT	<-->	WE	0.643	0.086	7.456	***
NCM	<-->	WE	0.500	0.074	6.791	***
NRL	<-->	WE	0.551	0.094	5.884	***

*N* = 233. CE, corporate entrepreneurship ecosystem; NAT, need for autonomy; NCM, need for competence; NRL, need for relatedness; WE, work engagement. *** *p* < 0.001.

We used gender and education level as control variables. Gender can determine needs and preferences with regard to job and workplace, which can affect engagement (Shah et al., 2025; Vuong and Suntrayuth, 2019). Education level can determine the expectations from the job and satisfaction of psychological needs.

## 4 Results

### 4.1 Results from qualitative study

The narratives from the qualitative empirical research have been explained using the lens of autonomy, competence, and relatedness of self-determination theory (SDT), which was used to structure the underpinned relationship between the CE ecosystem and millennials’ work engagement. The relationship between CE ecosystem, millennial work engagement, and needs of autonomy, competence, and relatedness were examined through thematic analysis. Table 3 includes the narrative text describing the impact of the CE ecosystem in terms of its five dimensions. The following explicates the results of analysis in terms of the hypothesised relationships.

**Table 3:** Thematic analysis of impact of CE ecosystem on work engagement of millennials.

Second order themes	First order categories	Evidence from representative data
Autonomy	Top management support	So, we create these enablers where employees can contribute, work hard, and work passionately. They’re given autonomy and also allowed to make some mistakes, okay. Then only they will learn something and they will learn it the hard way so that they do not make mistakes. P7
	Work discretion	We have gone way ahead and we say we do not have a performance management system, yearly. We have a continuous performance management system. So there is a lot of freedom given to the individual to make a difference. P1
	Rewards	Corporate entrepreneurship allows people to wear the entrepreneurship hat without quitting the company, without losing their monthly salaries. So with financial stability they are allowed to experiment within a safe environment which is the corporate environment. P6
	Time availability	The hackathons, there are live versions and on demand versions, you access it whenever you have the time, and you try to find a solution using your coding expertise. P10
	Organizational boundaries	We conceived a product and then it was like an innovative thing. So a lot of new people, who are part of the organization were given an opportunity to conceptualize and deliver, it kind of got launched also then it kind of cost effective product, which was made available to the farmers on apps. P3
Competence	Top management support	Today, there are organizations both large and small, which are either formally or informally supporting corporate entrepreneurship, especially with the current millennial generation because they look at more learning opportunities. It’s not just the money that matters, it’s also the culture that we provide to them also equally matters because what they are looking at is that they want to invest in a place where they can look, look at their own learning and learning and growth. P2
	Work discretion	They are happy with an assignment-based approach that you know, you give me a problem statement, I apply my competencies, I work and I learn from it. So I think all organizations are now realizing that no, that’s something that this workforce really aspires for. P3
	Rewards	If you want people to also show corporate entrepreneurship competencies, there has to be a substantial *financial reward* to it. Especially millennials and people who expect that because they have heard those concepts at college. P8
	Time availability	So the intricacies behind making the mask if he would not have invested his time and our managing director who inculcated this thought process or who channelized his energy as an employer if he would not have done we would not have achieved the competencies what we have achieved. P4
	Organizational boundaries	There is a lot of freedom given to the individual to make a difference and I deal with scientists and I’m sure you will understand, you know, scientists do not work in a compartment right. So there is no compartment for them. P3
Relatedness	Top management support	Keeping activities or team building activities will actually help team members to interact with each other, know each other’s likes, dislikes, and just bond over things which may or may not be related to work. P5
	Work discretion	I can write macros in Excel, reduce turnaround time on certain things we do, can automate things, but whether I do or I do not do, it’s my individual discretion. Nobody can mandate, nobody can push me to do it. That engagement comes only when the ecosystem in which I’m operating is something that I’m happy with and I feel connected to the place. P6
	Rewards	The financial support he extends to people irrespective of their level and display of their outfit. So that clearly sends the message that yes, he is there to take care, you do not have to worry. You are a part of the family. That belongingness he creates. P6
	Time availability	So they have to remain firm, have some patience, learn and prepare a clear agenda, nobody’s stopping. You can always inform your manager sir I’m here for 3 years and if I get this whatever exposure or *the training* , I will continue otherwise I will leave. P7
	Organizational Boundaries	This also helps for cross functional teams to get together in problem solving or in design thinking to come up with the right problem statements. P10

The table presents the main themes and sub-themes generated through NVivo coding, along with indicative quotations. The second-order themes (autonomy, competence, relatedness) were derived from self-determination theory, while first-order categories reflect CE ecosystem dimensions used as provisional codes and refined inductively during analysis.

Across the 10 HR professionals, analysis generated three overarching themes aligned with self-determination theory—autonomy, competence and relatedness—which were further organized around the five CE ecosystem dimensions (top management support, work discretion, rewards, time availability and organizational boundaries). In total, all 10 participants contributed to at least one code under autonomy, nine participants to competence, and eight participants to relatedness. Autonomy was the most frequently coded theme (62 coded references), followed by competence (49 coded references) and relatedness (37 coded references), indicating that respondents most often emphasized autonomy when describing how the CE ecosystem engages millennials.

The qualitative findings reported below are grounded in a systematic NVivo-based thematic analysis. First, CE ecosystem–related text was coded to the five CE dimensions, and then re-coded into three second-order themes corresponding to autonomy, competence and relatedness. Node statistics (number of respondents and coded references per theme) were used to identify dominant themes, while illustrative quotations were selected to represent views voiced by multiple participants rather than isolated comments.

#### 4.1.1 CE ecosystem and millennials engagement (Hypothesis 1)

Our analysis indicated that the CE ecosystem promotes work engagement of millennials as it facilitates appropriate rewards, sufficient time, tolerance of failure, and flexible organisational boundaries. Eight out of 10 participants explicitly linked CE initiatives to higher enthusiasm, commitment or retention among millennial employees. This pattern was particularly visible among participants from organizations with formal CE programmes, where CE was described as offering a “safe” space to innovate without sacrificing job security. For instance, P6, representing a manufacturing firm where approximately 50% of employees are millennials, noted that.

CE allows employees to *“*wear the entrepreneurship hat without quitting the company, without losing their monthly salaries. So where financial stability is taken care of, and they are allowed to experiment within a safe environment which is the corporate environment. When you give this kind of an opportunity, definitely people will be more engaged, and they would want to be there”, a sentiment echoed by six other participants who emphasized the combination of financial stability and experimentation as a key driver of engagement.

#### 4.1.2 Mediation of autonomy (Hypothesis 2)

Our analysis indicated that the CE ecosystem promotes work engagement of millennials by satisfying their need for autonomy. Participants mentioned that CE provides millennials decision-making latitude, desired freedom to offer new ideas and conduct experiments to accomplish projects. References to decision latitude, freedom to experiment and tolerance for mistakes were coded under autonomy; eight participants made such comments, with 19 coded references in total. CE also offers them liberty in actions and interactions with the environment. As P7 mentioned:

*I think the engagement of employees will go up as a lot of ideas are generated. So, employees feel that they are given autonomy to experiment and they are considered an integral part of the organization. P7.*

Moreover, participants mentioned that providing millennials employees with the discretion about carrying out their work, freedom from excessive oversight, encouragement to try new things as part of CE, promotes work engagement as the millennials feel that their needs for autonomy is satisfied.

*Rather than pushing an agenda of setting goals and holding the person accountable for deliverables, we have a continuous performance management system, this way a lot of freedom given to the individual to make a difference. P1.*

#### 4.1.3 Mediation of competence (Hypothesis 3)

Our analysis indicated that the CE ecosystem promotes work engagement of millennials by satisfying their need for competence. Seven out of 10 participants explicitly linked CE initiatives to enhancing the knowledge and skills among millennial employees. Participants mentioned that CE provides millennials, the chance, incentives, and recognition for executing challenging assignments, thus helping them enhance their knowledge and skills. References to incentives, and recognition for executing challenging assignments were coded under competence; seven participants made such comments, with 18 coded references in total.

CE offers an environment where they feel capable of dealing with the environment by using their competence. As P2 mentioned:

*With corporate entrepreneurship, there is a high probability of a higher employee engagement. I happened to be with this particular employee of ours who had created a new product, and he says that yes, I’m very happy because the kind of environment given to me, I was able to develop it and I’m looking at developing two or three more products for the company and that’s the reason he has been moved recently into the R&D teams.*

The participants further mentioned providing millennials employees as part of CE ecosystem with assignment-based approach where they apply their competence, giving free time to develop their competence promotes work engagement of millennials as they feel that their need for competence being satisfied.

*They are happy with an assignment-based approach where you give them a problem statement, they work by apply their competencies and learn from it. I think all organizations are now realizing that’s something that this workforce really aspires for. P3.*

#### 4.1.4 Mediation of relatedness (Hypothesis 4)

Our analysis indicated that the CE ecosystem promotes work engagement of millennials by satisfying their need for relatedness. Participants mentioned that CE gives millennials a sense of belonging, and enables them to develop trusted relationships with their colleagues and the organization. References to sense of belonging, and develop trusted relationships were coded under relatedness; six participants made such comments, with 18 coded references in total.

This pattern was particularly visible among participants from organizations with formal CE programmes, where CE was described as offering a “feeling of care” space where their ideas are given consideration by the organization. For instance, P7, representing an Infrastructure services firm where approximately 60% of employees are millennials, noted that *CE* makes millennials feel that they are cared for, as their ideas are given consideration by the organization.

*By practising corporate entrepreneurship definitely employee engagement will go up. There’s no doubt because the trust which the employees need to have with the employer and vice versa is something very important and the second thing is a lot of ideations. So, employees feel that they are considered, they are an integral part of the organization.* P7.

The participants further mentioned about offering millennials employees with financial support and rewards; offering millennials employees with team building activities, interaction opportunities where they interact with team members as part of CE ecosystem helps them create a team bond. All this promotes work engagement of millennials as they feel that their need for relatedness being satisfied.

*Keeping activities or team building activities actually help team members to interact with each other, know each other's likes, dislikes, and just bond over things which may or may not be related to work. P5*

*The financial support extended to people irrespective of their level clearly sends the message that yes, he is there to take care, you don't have to worry, that belongingness he creates. P6*

### 4.2 Results from quantitative study

The means, standard deviation and correlations are provided in Table 4. The inter-correlations among model variables were all statistically significant and positive. CE ecosystem demonstrated strong associations with need for autonomy (*r* = 0.69, *p* < 0.001), need for competence (*r* = 0.47, *p* < 0.001), need for relatedness (*r* = 0.66, *p* < 0.001), and work engagement (*r* = 0.66, *p* < 0.001). Need for autonomy correlated strongly with need for competence (*r* = 0.55, *p* < 0.001), need for relatedness (*r* = 0.64, *p* < 0.001), and work engagement (*r* = 0.68, *p* < 0.001), and need for competence correlated significantly with need for relatedness (*r* = 0.44, *p* < 0.001) and work engagement (*r* = 0.58, *p* < 0.001). Finally, the need for relatedness and work engagement were positively correlated (*r* = 0.55, *p* < 0.001). Also, CE ecosystem was negatively correlated with education level (*r* = −0.34, *p* < 0.001). For all the variables, the square root of AVE was greater than correlations with other variables (Table 4), thus indicating DV of the variables (Fornell and Larcker, 1981).

**Table 4:** Means, standard deviation and inter-correlations.

S.no.	Variables	M	S.D.	1	2	3	4	5	6	7
1	Gender	0.67	0.50	1						
2	Education level	1.21	0.48	0.12	1					
3	CE ecosystem	5.09	1.03	−0.03	−0.34***	**(0.71)**				
4	Need for autonomy	5.54	1.07	−0.05	−0.22^**^	0.69^***^	**(0.86)**			
5	Need for competence	5.77	0.79	0.02	−0.06	0.47^***^	0.55^***^	**(0.80)**		
6	Need for relatedness	5.41	1.03	−0.04	−0.23^***^	0.66^***^	0.64^***^	0.44^***^	**(0.79)**	
7	Work engagement	5.66	0.99	−0.06	−0.16^*^	0.66^***^	0.68^***^	0.58^***^	0.55^***^	**(0.82)**

*N* = 233. *p < 0.05; **p < 0.01; ***p < 0.001. Two-tailed significance values for Pearson correlation coefficient are reported. The figures in brackets denote square root of AVE for the corresponding model variable. Bold values denote the square root of AVE (average variance extracted) for the corresponding model variables. AVE has been explained in the text.

The measurement model fit the sample data well with *χ*^2^ (541) = 1020.23 (*p* > 0.05), CMIN/df = 1.89, root-mean-square error of approximation (RMSEA) = 0.06, standardized root-mean-square residual (SRMR) = 0.05, comparative fit index (CFI) = 0.92, and incremental fit index (IFI) = 0.92 (Bagozzi and Yi, 2012).

We conducted hierarchical regression to understand the variance explained by the different variables and to test Hypothesis 1 proposing the direct relationship between CE ecosystem and WE. Results, as presented in Table 5, reveal that the model fit increased in significance with the inclusion of gender and education level [Model 1: *F* (2, 230) = 3.2, *p* < 0.05, ∆*R*^2^ = 0.03], CE ecosystem [Model 2: *F* (3, 229) = 61.24, *p* < 0.001, ∆*R*^2^ = 0.42], and satisfaction of the need for autonomy, competence and relatedness [Model 3: *F* (6, 226) = 49.18, *p* < 0.001, ∆*R*^2^ = 0.12]. The table also shows that the CE ecosystem was positively and significantly related to WE (Model 2: *β* = 0.69, *p* < 0.001). Thus, Hypothesis 1 was supported. Besides, NAT (Model 3: *β* = 0.25, *p* < 0.001) and NCM (Model 3: *β* = 0.28, *p* < 0.001) were significantly related to WE and NCM was insignificantly related to WE (Model 3: *β* = 0.08, *ns*).

**Table 5:** Results of hierarchical regression on work engagement.

Variables	Model 1	Model 2	Model 3
Gender	−0.04	−0.05	−0.04
Education	−0.16*	0.08	0.03
Corporate entrepreneurship ecosystem		0.69***	0.29***
Need for autonomy			0.25**
Need for competence			0.28***
Need for relatedness			0.08
*R* ^2^	0.03	0.45	0.57
Adjusted *R*^2^	0.02	0.44	0.56
∆*R*^2^	0.03	0.42	0.12
*F*	3.2*	61.24***	49.18***

*N* = 233. *p < 0.05; **p < 0.01; ***p < 0.001. Two-tailed significance values are reported. Standardised coefficients are reported.

Before testing for mediation Hypotheses 2, 3, and 4, we needed to choose between a partial mediation (direct path between CE and WE) and a full mediation model (No direct path between CE and WE). The fitment indices of the partial mediation model [*χ*^2^ (612) = 1123.35, *p* > 0.05] and full mediation model [*χ*^2^ (613) = 1126.02, *p* > 0.05] are provided in Table 6. As the indices were equivalent, and the difference in the models was insignificant [∆*χ*^2^ (∆df) = 2.67(1), *ns*], we selected the more parsimonious full-mediation model (Werner and Schermelleh-Engel, 2010). This is in contradiction with the results for CE in Model 3 of hierarchical regression (Table 5). An argument for choosing SEM results is that path analysis more explicitly considers the direct relationships between CE and SDT variables than hierarchical regression, suggesting greater reliability of SEM results.

**Table 6:** Goodness of fit indices for alternate mediation models.

Alternate models	Overall fitment	CMIN/df	RMSEA	SRMR	CFI	IFI
Partial mediation model	*χ*^2^ (612) = 1123.35, *p* > 0.05	1.84	0.06	0.05	0.91	0.90
Full mediation model	*χ*^2^ (613) = 1126.02, *p* > 0.05	1.84	0.06	0.05	0.91	0.90

*N* = 233. Partial mediation model: Direct paths between CE and NAT, NCM and NRL; NAT, NCM and NRL and WE; CE and WE. Full mediation model: Direct paths between CE and NAT, NCM and NRL; NAT, NCM and NRL and WE. CE, corporate entrepreneurship; NAT, need for autonomy; NCM, need for competence; NRL, need for relatedness; WE, work engagement. RMSEA, root mean square error of approximation; SRMR, standardized root mean square residual; CFI, comparative fit index; IFI, incremental fit index.

We tested Hypotheses 2, 3 and 4 using path and effects analysis. For effects analysis, we used 5,000 bootstrap samples and 95% confidence intervals, with non-inclusion of zero within the confidence interval’s lower and upper limits indicating significant effects (Shrout and Bolger, 2002). The results of path analysis for the structural model are provided in Table 7.

**Table 7:** AMOS results for structural model.

Paths	*β*	*p*-values
CE ecosystem -> Need for autonomy	0.91	<0.001
CE ecosystem -> Need for competence	0.60	<0.001
CE ecosystem -> Need for relatedness	0.80	<0.001
Need for autonomy -> Work engagement	0.44	<0.001
Need for competence -> Work engagement	0.32	<0.001
Need for relatedness -> Work engagement	0.17	<0.05
Gender -> Work engagement	−0.04	0.42
Education level -> Work engagement	0.03	0.51

*N* = 233. *β*, standardised regression coefficients.

The path analysis showed that CE was positively related to NAT (*β* = 0.91, *p < 0.*001) and NAT was positively related to WE (*β* = 0.44, *p <* 0.001), confirming the results of hierarchical regression (Table 5). The effects analysis indicated significant indirect effects of CE on WE through NAT (effects = 0.196, BootSE = 0.073, LLCI = 0.052, ULCI = 0.340). Thus, Hypothesis 2 was supported.

CE was positively related to NCM (*β* = 0.60, *p < 0.*001) and NCM was positively related to WE (*β* = 0.32, *p <* 0.001), confirming the results of hierarchical regression (Table 5). The effects analysis indicated significant indirect effects of CE on WE through NCM (effects = 0.135, BootSE = 0.041, LLCI = 0.060, ULCI = 0.220). Thus, Hypothesis 3 was supported.

**Figure 1:**
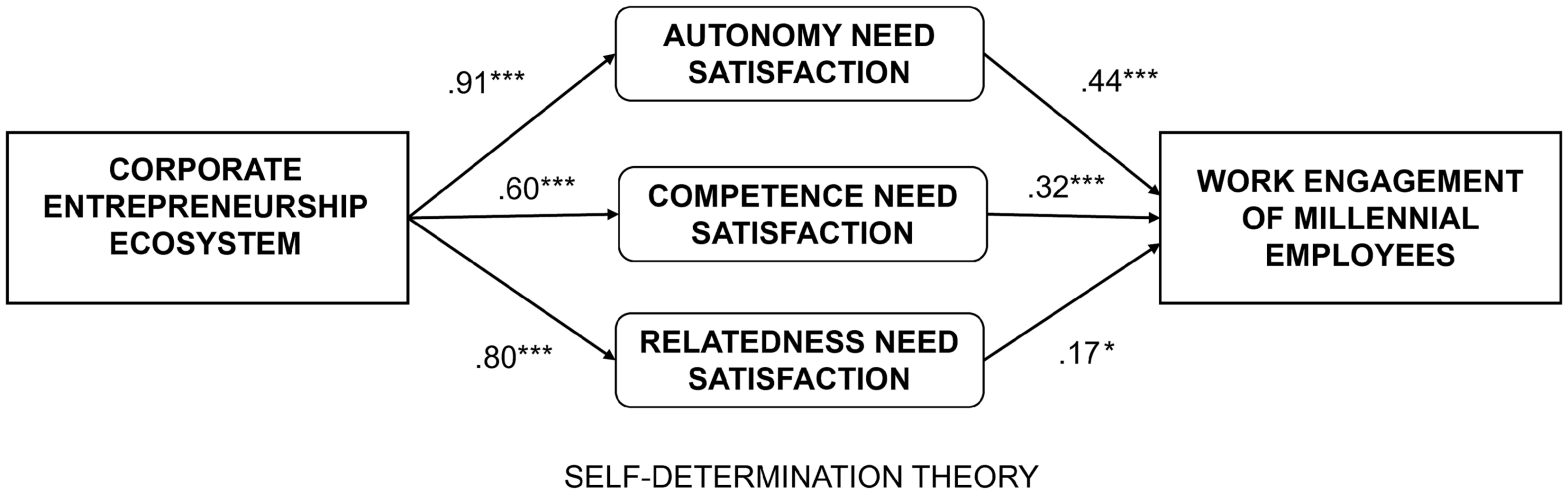
Results of path analysis.

CE was positively related to NRL (*β* = 0.80, *p < 0.*001) and NRL was positively related to WE (*β* = 0.17, *p < 0.*05). However, the indirect effect of CE on WE through NRL was insignificant (effects = 0.053, BootSE = 0.049, LLCI = −0.043, ULCI = 0.152). The results of hierarchical regression also showed an insignificant relation (Table 5). Since we received contradictory results through different forms of analysis, Hypothesis 4 could not be supported. Results of path analysis are presented in Figure 1.

To evaluate the effect size of the mediation variables, we considered Cohen’s *frerr^2^* (Cohen, 1988) and standardised indirect effects. The mediation of NAT, NCM, and NRL approached a large effect, as they collectively explained a substantial proportion of the CE–WE relationship, as corroborated by the incremental variance (Δ*R*^2^ = 0.12, *f*^2^ = 0.28) in Table 5. Standardised indirect effects (*β*₁ × *β*₂) for each hypothesised mediator (Preacher and Hayes, 2004) revealed that NAT demonstrated the largest mediation effect (standardised indirect effect = 0.40), NCM yielded a moderate indirect effect (standardised indirect effect = 0.19), whilst NRL produced a small, non-significant indirect effect (standardised indirect effect = 0.14).

## 5 Discussion

Our paper analyzes how the CE ecosystem engages millennial employees, and explores the role of SDT in determining this relationship. Through thematic analysis of interviews, and path and effects analysis of survey responses, the study reveals that the CE ecosystem leads to engagement of millennials by satisfying their need for autonomy and competence. Thus, autonomy and competence fully mediate the relation between the CE ecosystem and millennials WE. Relatedness, whilst theoretically plausible as a mediator, yielded inconclusive empirical results, a finding that warrants careful interpretation rather than dismissal. The similar results of qualitative and quantitative analysis also suggest that organizations are aligned to the employees in understanding that the CE ecosystem provides the autonomy and competence desired by millennials and thereby, engages them in their work. These findings contribute to interdisciplinary literature in entrepreneurship (for CE ecosystem) (Kuratko et al., 2014), positive psychology (for SDT) (Chhajer and Chaudhry, 2022; Spreitzer and Porath, 2014), and organizational behaviour (for engagement) (Wiedmer, 2015), and opens avenues for further cross-disciplinary research. The following paragraphs explain the contribution of the study.

The findings extend prior research on the CE and engagement relationship. Ahmed et al. (2020) established that CE promotes engagement as an antecedent of business performance, demonstrating the instrumental value of engagement within CE contexts. Our study advances this contribution by unpacking the psychological mechanisms, specifically autonomy and competence satisfaction, through which CE ecosystem initiatives translate into engagement, thereby moving beyond the demonstration of a CE - engagement relationship to explaining *why* and *how* this relationship operates. This mechanistic explanation represents a meaningful theoretical advance over Ahmed et al.'s (2020) outcome-focused perspective. Kassa and Raju's (2015) explanation of CE and engagement through social exchange theory (SET) perspective provides an important point of comparison. SET posits that employee engagement is driven by the perceived favourable balance of organisational benefits over individual costs, positioning engagement as a reciprocal behavioural response to organisational investment (Homans, 1958). Whilst this framework highlights the role of employees’ cognitive appraisal of CE benefits, it does not account for the intrinsic psychological processes through which CE ecosystem conditions sustain motivation independent of extrinsic cost–benefit calculations. Our SDT-based findings suggest that within CE ecosystems, autonomy and competence satisfaction engage employees at a more fundamental psychological level than the transactional exchange process described by SET.

The study’s primary theoretical contribution lies in explaining the relation between CE ecosystem and millennial engagement through the SDT lens. The findings suggest the fully mediating role of autonomy and competence. The full mediation finding is theoretically significant and warrants deeper interpretation beyond the mere confirmation of hypothesised paths. Full mediation implies that the CE ecosystem does not directly engage millennial employees. Rather, its motivational impact is entirely dependent on the satisfaction of autonomy and competence needs. This suggests that CE ecosystem initiatives that fail to meaningfully address these psychological needs, regardless of their structural perfection or resource utilization, are unlikely to produce intrinsic motivation and sustainable engagement outcomes among millennial employees. This interpretation aligns with SDT’s central proposition that the quality, rather than merely the quantity, of motivational conditions determines the depth and durability of work engagement, thus adding to the existing SDT literature (Gagné and Deci, 2005; Ryan and Deci, 2000; Spreitzer and Porath, 2014). The findings on the role of SDT in determining engagement also corroborate existing research examining their relationship (Hagger and McAnally Star, 2026; Liu et al., 2024; Nwoko and Yazdani, 2023). The implications of CE ecosystem also add to the literature on motivational antecedents of engagement (Byrne, 2022) and align with JD-R lens for explaining engagement (Faiz Rasool et al., 2024).

The full mediation of autonomy is consistent with prior SDT research demonstrating that autonomy-supportive organisational environments are among the strongest predictors of intrinsic motivation and engagement across diverse work contexts (Hagger and McAnally Star, 2026; Liu et al., 2024). Within the CE ecosystem specifically, autonomy satisfaction likely operates through the work discretion and time availability dimensions, which afford millennial employees the freedom to self-direct entrepreneurial activities, experiment with innovative ideas, and align their work behaviour with intrinsic values. These are the conditions that millennials are theoretically predisposed to find particularly engaging (Hartman and McCambridge, 2011).

The full mediation of competence similarly deepens the theoretical understanding of CE-driven engagement. Competence satisfaction within the CE ecosystem is likely facilitated through top management support and performance-contingent rewards, which provide millennial employees with the mentoring, resources, and affirming feedback necessary to develop mastery and confidence in entrepreneurial tasks (Kuratko et al., 2014; Deci et al., 1999). This finding corroborates existing SDT research linking competence satisfaction to sustained intrinsic motivation and engagement (Nwoko and Yazdani, 2023; Liu et al., 2024), whilst extending it to the specific organisational context of CE ecosystems.

Importantly, the mediation of both autonomy and competence (but not relatedness) suggests a nuanced motivational profile for millennial engagement within CE ecosystems, wherein job-related psychological needs for self-direction and mastery are more proximal drivers of engagement than interpersonal connectedness. This finding invites theoretical reflection on the boundary conditions of SDT within corporate entrepreneurship contexts, where task-oriented psychological needs may have greater motivational salience than relational needs.

The study makes an empirical contribution by exploring the relationship between the CE ecosystem, autonomy and competence, and work engagement among millennials in India. The study proposes and argues the hypothesised relationships, taking into account the distinctive characteristics, preferences and values of the millennials. This is the first study examining CE as a determinant of engagement for millennials. The study contributes to generational cohort theory (Trzesniewski and Donnellan, 2010) by indicating that millennials, due to their preference for meaningful, dynamic, and growth-oriented work (Hartman and McCambridge, 2011), can be especially responsive to CE. Also, by empirically understanding millennials’ engagement in the organizational context of CE ecosystem, the study adds to the existing literature on millennials’ engagement (Ali et al., 2022; Hui et al., 2021; Hurtienne et al., 2022; Sahni, 2021).

The study makes a methodological contribution by employing a mixed-methods design. Using a CPMM approach, we collected data through two different methods (qualitative and quantitative), from two different samples (HR managers and employees), and two different perspectives (organization and employees). This design allowed independent validation of the theoretical framework, mitigation of common-method bias, triangulation, and contextual and statistical understanding of results. It also helped verify complementarity of the findings and convergence of organizational and employee standpoints. Existing studies have not used mixed methods to understand the impact of CE on engagement.

The convergent parallel mixed-method design enabled systematic triangulation across methodological strands, producing a more comprehensive interpretation of findings than either method could achieve independently. Overall, qualitative and quantitative findings converged for Hypotheses 1, 2, and 3, whilst diverging for Hypothesis 4. For Hypothesis 1, hierarchical regression confirmed CE as a significant positive predictor of WE (*β* = 0.69, *p* < 0.001, Δ*R*^2^ = 0.42), corroborated by 8 out of 10 participants explicitly linking CE initiatives to enhanced millennial enthusiasm and commitment. Qualitative accounts contextualised this relationship by revealing that CE ecosystems engage millennials by creating psychologically safe entrepreneurial spaces that decouple innovation from personal financial risk, a motivational mechanism not directly captured by quantitative measurement. For Hypothesis 2, significant bootstrap indirect effects (effect = 0.196, 95% CI [0.052, 0.340]) were corroborated by 19 qualitatively coded references across eight participants, confirming that work discretion and reduced supervisory oversight within CE ecosystems satisfy millennials’ autonomy needs and translate into engagement. For Hypothesis 3, significant indirect effects (effect = 0.135, 95% CI [0.060, 0.220]) were supported by 18 coded references across seven participants, revealing that assignment-based entrepreneurial challenges constitute the primary competence-building mechanism. For Hypothesis 4, whilst bootstrap indirect effects were non-significant, six participants provided 18 coded references supporting relatedness mediation, indicating a divergence between quantitative and qualitative findings. However, thematic analysis revealed a bifurcation between organisational belonging and interpersonal connectedness suggesting a possible reason for non-significant mediation results.

Our findings were inconclusive for the mediation of relatedness due to conflicting quantitative results. Although path analysis showed a significant relationship between CE and NRL (*β* = 0.80, *p <* 0.001) and NRL and WE (*β* = 0.17, *p <* 0.05), the effects analysis did not indicate mediation (effects = 0.053, BootSE = 0.049, LLCI = −0.043, ULCI = 0.152). This inconsistency may be attributed to the relatively modest magnitude of the path coefficient between NRL and WE, which, despite reaching statistical significance, was insufficient to produce a significant indirect effect through bootstrapping (Preacher and Hayes, 2004). Additionally, the discrepancy between the significant individual paths and the non-significant indirect effect involving their product may reflect suppression effects (MacKinnon et al., 2002), where the portion of NRL variance attributable to CE does not correspond to the portion that drives WE, causing the indirect pathway to become non-significant. This may occur when the relatedness scale reflects more than one phenomenon. Thematic analysis for relatedness had revealed a bifurcation between organisational belonging and interpersonal connectedness (Table 3), suggesting that the CE ecosystems may relate to engagement through either of the aspects. This warrants use of a more nuanced and specific relatedness scale in future research. The non-significant mediation of relatedness may be associated with the contextual boundary conditions specific to CE ecosystems. Bakker and Demerouti’s (2007) JD-R theory suggests that the motivational salience of specific job resources varies according to the nature and demands of the work context. This may imply that entrepreneurial work environments, characterised by individual initiative, task autonomy, and performance accountability, prioritise task-oriented psychological needs, like autonomy and competence, over interpersonal relatedness in determining engagement outcomes.

### 5.1 Practical implications

We found that a CE ecosystem can intrinsically motivate millennials by satisfying their inherent psychological needs, leading to engagement that sustains over time. The quantitative analysis showed that the CE ecosystem was positively and significantly related to millennials’ work engagement and that this relationship was fully mediated by satisfaction of autonomy and competence needs. Path and effects analyses confirmed significant indirect effects of CE on engagement through autonomy and competence, whereas relatedness did not emerge as a significant mediator in the full model.

These results imply that organizations seeking to engage millennials should design CE ecosystems that deliberately enhance autonomy and competence rather than focusing only on generic engagement practices. Given that autonomy significantly mediated the CE–engagement relationship, organizations should prioritise CE practices that enhance decision latitude and freedom to experiment for millennials, such as project-based roles, flexible task design, participation in internal venture teams, and explicit tolerance for failure in innovation projects. These practices directly address the autonomy pathway identified in the structural model, and are consistent with qualitative accounts that emphasised freedom from excessive oversight and discretion in how work is carried out (e.g., participants describing continuous performance management and flexibility in task execution).

Because competence also significantly mediated the CE–engagement link, CE policies should incorporate challenging assignments, opportunities to lead internal entrepreneurial initiatives, and structured feedback and recognition mechanisms that build millennials’ efficacy. For example, respondents highlighted assignment-based approaches and opportunities to launch new products as particularly engaging for millennials, as these experiences allowed them to apply and develop their competencies and see tangible outcomes from their efforts. Such practices resonate with qualitative accounts of millennials seeking learning and growth through CE initiatives and satisfy the need for competence.

From a generational perspective, the findings show that CE ecosystems are especially effective for millennials when they align with this cohort’s preference for autonomy, continuous learning and meaningful collaboration. The qualitative results illustrate that HR heads perceive millennial employees as more engaged when CE initiatives allow them to “wear the entrepreneurship hat without quitting the company” and maintain financial stability (P6), when performance management systems emphasise ongoing feedback rather than rigid annual appraisals (P1), and when CE practices create opportunities for cross-functional collaboration and recognition of ideas (e.g., hackathons and team-based innovation projects). These insights suggest that CE policies tailored to millennials should combine internal venture opportunities with visible career progression pathways, rapid feedback loops, and a psychologically safe climate for experimentation.

Taken together, the empirical evidence leads to several concrete implications for organizations. First, they can adopt and strengthen a CE ecosystem as a strategic lever to attract, engage and retain millennial employees, especially in contexts where this cohort constitutes a dominant share of the workforce. Second, they can institutionalise supportive CE policies and processes, such as top management sponsorship, dedicated time for experimentation, and transparent reward systems, which explicitly aim to satisfy millennials’ needs for autonomy and competence through their everyday work roles. Third, in economies with low engagement rates and a large millennial workforce, enterprises can track the effectiveness of CE initiatives by regularly assessing perceived autonomy and competence, alongside traditional engagement metrics, and use this information to fine-tune CE initiatives design. Finally, as relatedness did not mediate the CE–engagement relation in the full model, organizations may prioritise autonomy and competence-enhancing features when resources are constrained, while still recognising that relatedness can add value by strengthening millennials’ sense of belonging to the organization, as suggested by the qualitative findings.

### 5.2 Limitations and research avenues

Testing of mediation of relatedness can be done for bigger sample sizes by including millennial employees from more industries and organizations. Besides, we can conduct a cross-generational analysis to see whether results for relatedness are generation-specific or follow a similar pattern across generations. Considering the qualitative results for mediation, we can also create separate measures for relatedness to organization and relatedness to peers, and check for their mediation between CE ecosystem and millennials engagement.

We collected data from limited Indian organizations. Future studies can gather data from more organizations of different industries, sizes, turnover and countries. The results would help understand whether our hypotheses are supported irrespective of the context, and what the variations in the findings across contexts are, if any. Moreover, the intervening effects of autonomy, competence, and relatedness can be explored with regard to organizational factors like transformational leadership, leader-member exchange, organizational culture, perceived organizational support, high-performance work systems, learning and development and performance management. Further, we can test the entire model for Generation Z and Generation X employees, to investigate if the relation between CE ecosystem, engagement, and SDT differs across generations.

The use of purposive sampling for the qualitative phase and snowball sampling for the quantitative phase limits the generalisability of the findings. The samples may over-represent organisations and employees that are more positively disposed towards CE or more engaged with CE initiatives. Future research could employ probability-based sampling or use multi-organisational datasets with larger and more diverse employee samples to confirm the robustness of the relationships observed here.

## Data Availability

The raw data supporting the conclusions of this article will be made available by the authors, without undue reservation.

## Appendix

Semi-structured interview guide with itemised questions.

- “Can you describe the key features of corporate entrepreneurship initiatives in your organization?”
- “How, if at all, do these initiatives affect the way millennial employees feel about their work?”
- “In what ways do CE initiatives influence the freedom or autonomy that millennials experience at work?”
- “Can you share an example where a CE initiative helped a millennial employee develop new skills or feel more competent?”
- “How do CE practices shape relationships, collaboration or sense of belonging among millennial employees?”
